# Mobile phones: influence on auditory and vestibular systems

**DOI:** 10.1016/S1808-8694(15)30762-X

**Published:** 2015-10-19

**Authors:** Aracy Pereira Silveira Balbani, Jair Cortez Montovani

**Affiliations:** 1 PhD, Voluntary Adjunct Professor, Otorhinolaryngology and Head & Neck Surgery Discipline, Botucatu Medical School (UNESP); 2 Associate Professor (livre-docente habilitation), Otorhinolaryngology and Head & Neck Surgery Discipline

**Keywords:** hearing, microwaves, radio waves, cellular phone

## Abstract

Telecommunications systems emit radiofrequency, which is an invisible electromagnetic radiation. Mobile phones operate with microwaves (450900 MHz in the analog service, and 1,82,2 GHz in the digital service) very close to the user's ear. The skin, inner ear, cochlear nerve and the temporal lobe surface absorb the radiofrequency energy.

**Aim:**

literature review on the influence of cellular phones on hearing and balance.

**Study design:**

systematic review.

**Methods:**

We reviewed papers on the influence of mobile phones on auditory and vestibular systems from Lilacs and Medline databases, published from 2000 to 2005, and also materials available in the Internet.

**Results:**

Studies concerning mobile phone radiation and risk of developing an acoustic neuroma have controversial results. Some authors did not see evidences of a higher risk of tumor development in mobile phone users, while others report that usage of analog cellular phones for ten or more years increase the risk of developing the tumor. Acute exposure to mobile phone microwaves do not influence the cochlear outer hair cells function in vivo and in vitro, the cochlear nerve electrical properties nor the vestibular system physiology in humans. Analog hearing aids are more susceptible to the electromagnetic interference caused by digital mobile phones.

**Conclusion:**

there is no evidence of cochleo-vestibular lesion caused by cellular phones

## INTRODUCTION

**Figure 1:**
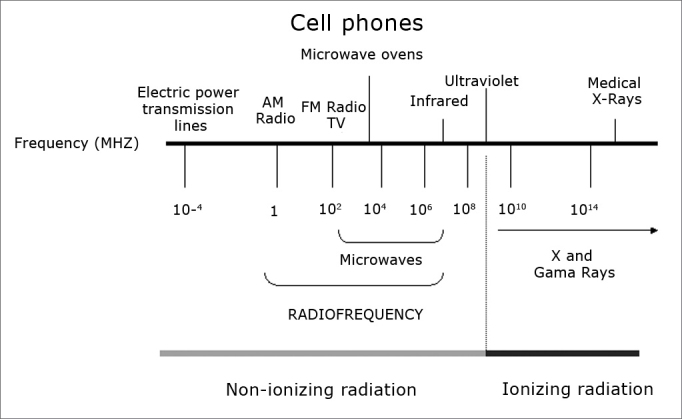
Electromagnetic radiation frequency spectrum.

Telecom systems - radio, television, wireless telephones, mobile phones, pagers, radars and satellites - emit invisible electromagnetic radiation or radiofrequency (RF). The radiation spectrum includes microwaves (frequencies between 300 MHz and 300 GHZ) and reaches close to infrared radiation.^1^^,^^2^ (Figure 1)

RF is also used daily in microwave ovens and diathermy medical devices (thermoablation); the latter are used in treating cardiac arrhythmias, tumors and other conditions.^3^^,^^4^

Since the 1970s, the World Health Organization, governments of various countries, researchers, and manufacturers of telecom equipment have studied the effects of RF on human health. The large number of radio transmitters and telecom antennae installed close to residential areas and the growing use of mobile phones justify these efforts.^5^ Based on these studies, regulations have defined tolerance levels for human exposure to electromagnetic radiation, which technical committees review periodically.^1^^,^^5^

There are still, however, concerns about the possibility of lymphatic cancer, central nervous system tumors (including acoustic neuromas), choroidal melanomas, and other conditions in subjects chronically exposed to RF, which have motivated epidemiological and experimental studies.^5^, ^6^, ^7^, ^8^

One of the most frequently discussed themes currently is the effect of mobile phone use on human health, given that these devices transmit microwaves (450–900 MHz in analog systems, and 1.8–2.2 GHz in digital systems)^5^ very close to a user's heads, specifically to the ear. Brazil currently has about 90 million mobile phone users.^1^

This review presents themes of interest for otorhinolaryngologists, such as the biological effect of RF, the relation between mobile phone use and central nervous system tumors, and the effects of mobile phone electromagnetic radiation on the auditory system, on the vestibular labyrinth, and on hearing aids.

## REVIEW OF THE LITERATURE

Lilacs and Medline database indexed papers were reviewed. We selected papers published from 2000 to 2005. We also reviewed published matter from the National Telecommunications Agency (Agencia Nacional de Telecomunicacoes) - ANATEL, and Internet website materials from mobile phone manufacturers.

### Biological effects of radiofrequency/microwaves

RF is a non-ionizing radiation, as opposed to X-rays and gama radiation; it does not, therefore, have enough energy to destabilize electrons or break chemical bonds in DNA.^2^^,^^5^^,^^9^

The effect of RF on living organisms may be didactically divided into the following:

1) thermal effects: these are the best known effects. They result from water molecule polarization as electromagnetic waves course through tissues and produce heat (temperature variation over 1oC).^9^ This is the principle behind microwave ovens and medical diathermy devices.^4^
  Devices that generate RF between 350 and 500 kHz or microwaves over 2 GHz are used for thermoablation surgery. Tissue temperatures reach 50–100oC, resulting in local necrosis and coagulation. Temperatures over 100oC vaporize and carbonize tissues.^3^
  Telecom workers that are accidentally exposed to high RF loads absorb this energy, which produces heat. They may have skin burns and injury to heat-sensitive tissues, such as the lens of the eye, the testicles and the brain, leading respectively to cataract, male infertility and seizures.^1^^,^^5^ Safety guidelines are therefore needed for screening RF/microwave-emitting devices and for protecting workers that may be exposed to this radiation.^1^
  The power of radio and television transmitters may reach many kilowatts; mobile phone base stations may reach over 100 W.^5^^,^^10^ On the other hand, the power of mobile phone handsets and cordless phone base units is very low, respectively around 0.01–2 W and 0.09W.^5^^,^^10^^,^^11^ For this reason mobile phones do not cause thermal effects on a user's organisms. It has been calculated that the temperature in the head increases by not more than 0.11oC5 while using a mobile phone, although a feeling of warmth may be felt in the ear during a telephone call.^12^
2) non-thermal effects: these take place with no temperature change in biological tissues. These effects have not yet been fully clarified, and are the reason for many debates among scientists.

These effects include electrical force induction and possibly an increase in heat shock protein synthesis in cells.^13^ The most significant expression of these proteins occurs in the physiological cell defense response against oxidative stress and in osmotic pressure variations, among other factors. Continuous heat shock protein synthesis, however, may be involved in oncogenesis, by inhibiting cell apoptosis.^13^ This mechanism might explain how chronic exposure to high RF loads could cause cancer in susceptible subjects,^13^ a mechanism that is still under debate in the scientific community.

No study thus far has demonstrated that exposure to RF without thermal effects produces genetic mutations or chromosomal aberrations in mammal cells, which suggests that RF cannot initiate tumors.^2^^,^^9^

D'Andrea et al. (2003)^14^ have reviewed studies about the possible non-thermal effects of mobile phone electromagnetic radiation on the electrical activity of the central nervous system (CNS). Results are controversial; some studies have found no electroencephalographic changes, while other have reported alterations in alpha and beta waves in humans, and delta waves in rats.^14^

### Measurement of the radiofrequency dose

The specific absorption rate (SAR) is used to measure the energy dose that subjects exposed to RF absorb. The SAR is expressed in power (Watts) by tissue mass (kilograms).^1^^,^^2^

The full body SAR in human beings depends on various factors, such as: the nature of an electromagnetic field (low or high frequency); the distance and spatial orientation of the field relative to a subject; the subject's geometry (whether a baby, a child or an adult, or whether tall or short); and the water content of different tissues.^1^

The SAR also varies according to the mobile phone handset model, the transmission system technology (analog or digital), the distance between a user's head and the handset antenna, and the distance between a mobile phone and its base station.^2^

Digital handsets (TDMA - time division multiple access, CDMA - code division multiple access, and GSM - Global System for Mobile Communications) expose a user's head to a SAR almost always below 1 W/kg, well below the recommended maximum safe exposure limits.^2^^,^^11^ Radiological protection committees from various countries have tended to set the SAR tolerance limit at 10 W/ kg.^15^ It is important, however, to follow the manufacturer's recommendations, such as not touching the antenna or not allowing it to touch the head while using a handset.

### Relation between mobile phone use and central nervous system tumors

While using a mobile phone, the skin around the ear, the inner ear, the vestibulocochlear nerve, and a small surface area of the temporal lobe absorb RF and microwave energy.^16^ Hypothetically, these areas would be at a higher risk for tumors in subjects that use mobile phones regularly.

A number of case-control studies have investigated the relation between mobile phone use and CNS tumors (Table 1).

**Table 1:** Case-control studies on the risk of central nervous (CNS) tumors due to mobile phones.

	Sample	Relative risk (RR) for tumor (Confidence Interval [CI] – 95%)	Conclusion
Muscat et al. (2000)^17^	469 cases 422 controls	RR 0.7 (CI – 95% = 0.3–1.4)	No increased risk
Inskip et al. (2001)^18^	782 cases: 96 acoustic neuromas, 799 controls	RR 0.9 (CI 95%= 0.7–1.1) for all tumors	No increased risk
		RR 0.8 (CI 95%= 0.5–1.4) for acoustic neuromas	
Muscat et al. (2002)^19^	90 acoustic neuromas 86 controls	RR 0.9 (CI 95%= 0.3–1.4)	No increased risk
Hardell et al. (2003)^20^	1429 cases 1470 controls	RR 1.3 (CI 95%= 1.04–1.6) for analog handsets RR 1.4 (CI 95%=0.9–2.1) for digital handsets - use>5 years	Increased risk when using analog handsets, and when using digital handsets for over 5 years
Christensen et al. (2004)^21^	106 acoustic neuromas 212 controls	RR 0.9 (CI 95%= 0.51–1.57)	No increased risk
Lönn et al. (2004)^7^	148 acoustic neuromas 604 controls	RR 1.9 (CI 95%= 0.8–4.3) for analog handsets	Increased risk when using analog handsets for 10 years or more
Hardell et al. (2005)^23^	413 cases: 84 acoustic neuromas, 692 controls	RR 4.2 (CI 95%= 1.8–10) for analog handsets RR 8.4 (CI 95% 1.6–45) for handset use >15 years	Increased risk when using analog handsets

Muscat et al. (2000)17 analyzed 469 cases of cerebral neoplasms and 422 controls. The mobile phone use profile was similar in both groups; males aged 30 to 49 years predominated, most of which worked as salespersons. No association between exposure to mobile phone-generated RF and neoplasms was found. Furthermore, temporal lobe tumors were more likely to arise on the opposite side to that where mobile phones were habitually used.

Inskip et al. (2001)18 and Muscat et al. (2002)19 reported similar results. No relation was found between tumor diagnosis, the duration of handset use (in years) and the frequency of telephone calls.

These three studies collected data from the 1990s, when most of the handsets were analog. These have been replaced by digital technology, where microwave emissions have a lower output power at higher frequencies.^2^ Hardell et al. (2003)^20^ took this variable into account and analyzed 1,429 cases of CNS tumors. These authors found that analog handsets used for over one year, and digital handsets used for over five years, led to an increased risk of tumors, mostly temporal lobe tumors. There were 159 acoustic neuroma cases included in this paper, for which the relative risk (RR) was 4.4 (confidence interval [IC] 95% = 2.1–9.2) in analog handset users, and 1.4 (CI 95% = 0.8–2.4) in digital handset users; in other words, there was a higher than expected probability of subjects developing these tumors.

Christensen et al. (2004)^21^ analyzed acoustic neuroma cases and found no increased risk for tumors in mobile phone users. These authors also found no relation between using a mobile phone for 10 years or more and the incidence of tumors, between using the handset mostly on the right or the left and the tumor site, or duration of exposure to handset radiation and neuroma size.

Lönn et al. (2004)^7^ investigated 148 acoustic neuroma cases and found a relation between tumor occurrence and use of an analog handset for 10 years or more, with a preference for the ear that was most irradiated. Failures in the statistical analysis were later pointed out in this study.^22^

Hardell et al. (2005)^23^ studied 413 patients that had benign CNS tumors (305 meningioma cases, 84 acoustic neuroma cases, and 24 other histological types) and found an increased risk in analog handset users. The RR was even higher for subjects that had used mobile phones for over 15 years. Although the series was small, the authors concluded that mobile phone use was a risk factor for the occurrence of acoustic neuroma.

Johansen et al. (2001)^24^ analyzed data from 420,095 mobile phone users in a Danish retrospective cohort study. The RR for CNS tumors in these subjects was 0.95 (CI 95% = 0.8–1.1). The RR for temporal lobe tumors was 0.86 (CI 95% = 0.4–1.5). There was no increased risk for acoustic neuromas. A caveat of this study is that 69% of the subjects had used mobile phones for only 1–2 years, which makes it difficult to evaluate the impact of prolonged mobile phone use on the occurrence of CNS tumors.^2^

An important point to consider when interpreting this data is that the duration of use (in years) and frequency of use (number or duration of calls) of mobile phone are imprecise measures of microwave exposure. As we saw in the section on biological effects cause by RF, the SAR will vary significantly depending on many factors.^2^

### Effects of mobile phone electromagnetic radiation on the auditory system

#### 1) Short-term effects

Ozturan et al. (2002)^11^ assessed transient and distortion product evoked otoacoustic emissions in 30 normal-hearing adults before and after a 10-minute telephone call using a GSM mobile phone transmitting microwaves at 900 MHz. Otoacoustic emissions did not change as a result of using a mobile phone.

Oysu et al. (2005)^25^ assessed the short-term effects of mobile phone electromagnetic fields on the auditory evoked potential (BERA) of 18 normal-hearing volunteers. The handset transmitted microwaves at 900 MHz, the SAR was 0.82 W/kg, and the position was the right ear. BERA was done before and immediately after a 15-minute telephone call. The authors found no significant variation in waves I, II and V, or in the interpeak intervals I-III, III-V and I-V.

Sievert et al. (2005)^26^ tested the influence of continuous or pulsed microwaves on the BERA of 12 healthy volunteers. The test was done before, during and after using a mobile phone. Handsets transmitted microwaves at 889 MHz and were used alternatively on both ears. There were no significant changes in BERA from using mobile phones.

Aran et al. (2004)^8^ cultured Corti organ cells from newborn rats in an in vitro experiment, exposing these cells to GSM microwaves at a dose of 1 W/kg for 24–48 hours, and found no changes in hair cell ultrastructure.

#### 2) Long-term effects

Kizilay et al. (2003)^15^ investigated possible interferences of RF on the cochlear physiology of rats. Distortion product otoacoustic emissions were recorded in newborn and adult animals exposed to GSM microwaves at 900 MHz during one hour each day for 30 days. These animals were compared to an unexposed control group. No changes in otoacoustic emissions were found in growing and adult rats. The authors hypothesized that the compact bone around the cochlea might have shielded it against radiation, protecting the hair cells from microwaves.

Aran et al. (2004)8 conducted an experimental model using guinea pigs exposed to GSM microwaves at 900 MHz in one ear only for one hour each day, five days a week, during two months,. The guinea pigs were divided into four groups: exposure to a SAR of 1 W/kg, of 2 W/kg, of 4 W/kg, and non-exposed (controls). Distortion product otoacoustic emissions and BERA thresholds were assessed before, during and after the experiment. Finally, the animals were sacrificed for histological studies of the cochlea.

No dose-response effects of radiation on otoacoustic emissions were found. BERA revealed a significant mean threshold increase in both ears of every animal (exposed to microwaves and controls) during the two months that the experiment lasted. The authors explained these findings describing that as the animals grew, their heads increased, and so did the distance between central auditory pathways and the auditory evoked potential recording electrodes. There were no threshold differences between the radiation-exposed and the contralateral ear (non-exposed) in microwave-irradiated animals.^8^

Histology revealed complete ossification of an irradiated cochlea in only one guinea pig (in the SAR 2 W/kg group) out of 32 animals. According to the authors, unilateral ossifying labyrinthitis is common in guinea pigs due to otitis media and bacterial meningitis; so this finding cannot be attributed exclusively to radiation.^8^

### Effect of mobile phone electromagnetic radiation on the vestibular system

Pau et al. (2005)^27^ did computerized nystagmography on 13 volunteers with no vestibular diseases that were under the effect of pulsed or continuous 900 MHz microwaves. Minor amplitude nystagmus was detected in five subjects during exposure to continuous microwaves, and in four subjects during exposure to pulsed microwaves. These findings, however, were considered insignificant, and not a vestibular response.

The authors also conducted an infrared thermography experiment on bone and soft tissues to assess any eventual thermal effect produced by mobile phones on the lateral semicircular canal. No temperature variations were found at a depth that corresponded to that of the labyrinth, suggesting that mobile phone microwave transmitting power is not sufficient to cause heating.^27^

### Effects of mobile phone electromagnetic radiation on hearing aids

Older hearing aids - particularly analog devices - may be susceptible to electromagnetic interference. Pulsed microwaves, used in digital handsets, may be demodulated by hearing aid electric circuit semiconductors, resulting in noise.^28^^,^^29^

A mobile phone handset manufacturer offers an accessory that may be used by subjects using analog prostheses. It is a loop used around the neck that transmits mobile phone signals to the hearing aid by induction. According to this manufacturer, the induction loop improves the user's acoustic comfort.^29^

## DISCUSSION

Technological developments in telecommunications systems have brought undeniable benefits; possible harmful effects of RF and microwaves, however, are still a controversial issue.

Published papers are based on North-American and European research - particularly from Scandinavian countries - the home of major world manufacturers of mobile telecommunication handsets and base stations. No evidence has shown any causal relation between RF exposure and the occurrence of cancer, CNS tumors, or other diseases.^2^^,^^16^

The International Commission on Non-Ionizing Radiation Protection has stated that nearly all of the epidemiological surveys have focused on adults.^5^ The effects of RF and microwaves on children and teenagers, who currently are frequent users of mobile phones, are still unknown.^5^ Furthermore, if we admit that RF and microwaves may take decades to initiate tumors, monitoring people exposed to radiation will have to continue before we conclude that there is no risk for developing neoplasms.^5^^,^^16^

There is no evidence supporting the influence of mobile phone use on the occurrence of benign or malignant CNS tumors, especially those located in the temporal lobe.^17^^,^^18^^,^^24^ Results of studies on mobile phone handset radiation and the risk of developing acoustic neuroma have been contradictory (Table 1). Some authors have found no increased probability of tumor development in mobile phone users,^18^^,^^19^^,^^21^^,^^24^ while other have stated that the use of mobile phones - particularly analog handsets - for 10 years or more is a risk factor for developing tumors.^7^^,^^20^^,^^23^

Muscat et al. (2002)^19^ have pointed out that acoustic neuromas are slow-growing tumors, and may have been present in case-control study subjects before they began to use mobile phones. A further point is that cranial nerve tumors are rare, and the neuroma cases that were studied were part of small series.^7^^,^^18^, ^19^, ^20^, ^21^^,^^23^^,^^24^

Various papers have suggested that exposure to mobile phone microwaves has no influence on the activity of cochlear outer hair cells or of cochlear nerve electrical conduction, both in vivo and in vitro.^8^^,^^11^^,^^15^^,^^25^^,^^26^ Another point is that all of the studies on humans were carried out on normal-hearing volunteers. It is not know whether the cochlea of patients with inner ear conditions would be more sensitive to electromagnetic radiation.

Notwithstanding the evidence, Ozturan et al. (2002)^11^ have recommended caution in mobile phone use to minimize exposure of the auditory system to microwaves. These authors have proposed that mobile phones should be used only when absolutely needed, and even then, for short calls. Hands-free kits are preferred, as they may reduce the microwave load by about 90%.^7^

Apparently, mobile phone microwaves have no short-term effects on the vestibular system of normal subjects.^27^ There are, however, no data in the literature on the influence of chronic exposure to this type of radiation.

These unresolved issues justify future studies to better understand the effect of mobile phone electromagnetic radiation on the auditory and vestibular systems; this includes the risk of tumors in the eighth cranial nerve. Any adverse effect that may eventually be found should be promptly reported; it is a health issue of interest to billions of users worldwide.

Currently manufactured digital hearing aids include effective protective features against mobile phone electromagnetic interference.^28^ Subjects still using analog prostheses may couple their handsets to an induction loop, albeit at a cost. User of any hearing aid may, thus, use digital mobile phones with no discomfort.

## FINAL COMMENTS

There is no scientific evidence suggesting that RF/microwaves emitted by mobile phones cause thermal harm to users.

Acute exposure to microwaves transmitted by mobile phone handsets does not affect the cochlear outer hair cell activity, the cochlear nerve electric conduction or vestibular labyrinth function in vivo or in vitro.

There are controversial data about an increased risk of acoustic neuroma in chronic mobile phone users.

Analog hearing aids are more prone to electromagnetic interference caused by digital mobile phone handsets.

Finally, the mobile phone electromagnetic interference on implanted cardiac pacemakers and medical/hospital equipment (heart monitors, infusion pumps, pulse oxymeters, non-invasive arterial blood pressure monitors, etc.) should be noted. Just as mobile phones are required to be switched off in aircraft, it is highly recommended that doctors refrain from using mobile phones in operating rooms and intensive care units, for the safety of patients. In certain situations, mobile phone electrical fields may overwhelm the shielding capability of electrical medical devices, causing them to switch off, setting off alarms irregularly or yielding incorrect vital sign measurements.^30^

## ACKNOWLEDGMENTS

We wish to acknowledge Mr. Jose Amaro O. Balbani for kindly clarifying the technical aspects and for strongly encouraging this study.
